# Extracellular Matrix and Growth Factors Improve the Efficacy of Intramuscular Islet Transplantation

**DOI:** 10.1371/journal.pone.0140910

**Published:** 2015-10-16

**Authors:** Haruyuki Tsuchiya, Naoaki Sakata, Gumpei Yoshimatsu, Masahiko Fukase, Takeshi Aoki, Masaharu Ishida, Yu Katayose, Shinichi Egawa, Michiaki Unno

**Affiliations:** 1 Department of Surgery, Tohoku University, Sendai, Japan; 2 Division of Integrated Surgery and Oncology, Tohoku University Graduate School of Medicine, Sendai, Japan; 3 Division of International Cooperation for Disaster Mediscine, International Research Institute of Disaster Science, Tohoku University, Sendai, Japan; Osaka University, JAPAN

## Abstract

**Background:**

The efficacy of intramuscular islet transplantation is poor despite being technically simple, safe, and associated with reduced rates of severe complications. We evaluated the efficacy of combined treatment with extracellular matrix (ECM) and growth factors in intramuscular islet transplantation.

**Methods:**

Male BALB/C mice were used for the *in vitro* and transplantation studies. The following three groups were evaluated: islets without treatment (islets-only group), islets embedded in ECM with growth factors (Matrigel group), and islets embedded in ECM without growth factors [growth factor-reduced (GFR) Matrigel group]. The viability and insulin-releasing function of islets cultured for 96 h were significantly improved in Matrigel and GFR Matrigel groups compared with the islets-only group.

**Results:**

Blood glucose and serum insulin levels immediately following transplantation were significantly improved in the Matrigel and GFR Matrigel groups and remained significantly improved in the Matrigel group at postoperative day (POD) 28. On histological examination, significantly decreased numbers of TdT-mediated deoxyuridine triphosphate-biotin nick end labeling-positive islet cells and significantly increased numbers of Ki67-positive cells were observed in the Matrigel and GFR Matrigel groups at POD 3. Peri-islet revascularization was most prominent in the Matrigel group at POD 14.

**Conclusions:**

The efficacy of intramuscular islet transplantation was improved by combination treatment with ECM and growth factors through the inhibition of apoptosis, increased proliferation of islet cells, and promotion of revascularization.

## Introduction

Islet transplantation has demonstrated utility as a cell replacement therapy for severe diabetes mellitus (DM). Clinical islet transplantation has been predominantly performed using an intraportal approach. However, there are several issues associated with using the liver as a transplant site. A majority of intrahepatic transplanted islets fail to engraft for a number of reasons, including instant blood-mediated inflammatory reaction [1], the influence of immunosuppressants [2], and excessive hepatic lipogenesis [3]. In addition, intraportal transplantation is associated with severe complications such as portal hypertension and embolism [4], which can be life threatening [5].

A number of organs have been studied as alternative transplantation sites for islet cells, including the kidney [6], greater omentum [7], bone marrow [8], and pancreas [9]; however, these sites are regarded as suboptimal transplantation sites in clinical settings. The muscle has also been considered a candidate transplant site for islets. Intramuscular transplantation is technically simple and safe, and it confers a decreased risk of severe complications. However, the efficacy of intramuscular transplantation is low because of poor oxygen tension and blood supply due to a lack of early neovascularization following transplantation [10]. Thus, modifications to currently used protocols are required to improve the efficacy of intramuscular transplantation prior to its application in the clinical setting.

With an aim to improve the efficacy of intramuscular islet transplantation, we evaluated the use of extracellular matrix (ECM) and growth factors. ECM is a noncellular component that initiates crucial biochemical and biomechanical cues required for tissue morphogenesis, differentiation, and homeostasis in addition to providing essential physical scaffolding for cellular constituents [11, 12]. Growth factors are naturally occurring substances capable of stimulating cellular growth, proliferation, healing, and cellular differentiation [13–15]. Previous studies have demonstrated the efficacy of ECM and growth factors. Rackham *et al*. reported transplanted islets embedded in ECM have rich vascularization and good endocrinal function in a mouse model [16]. Edamura *et al*. demonstrated successful bioartificial pancreas transplantation using ECM in a pig to dog xenotransplant model [17]. Regarding growth factors, our recent study demonstrated nerve growth factor (NGF) induction improved neovascularization in transplanted islets and resulted in normalization of blood glucose (BG) levels in diabetic mice [18]. Another study also revealed that vascular endothelial growth factor (VEGF) derived from co-transplanted bone marrow (mesenchymal stem) cells contributed to the neovascularization of transplanted islets and improvement of the endocrinal condition [19]. We hypothesize that combination treatment of ECM and growth factors will facilitate the engraftment of intramuscularly transplanted islets, improve transplant efficacy, and lead to clinical application in the future.

In this study, we chose Matrigel as a representative ECM agent. Matrigel consists of various ECMs. For example, laminin, a major component of Matrigel, has been posited as important for β-cell proliferation and insulin transcription [20]. Collagen IV has been shown to contribute to the regulation of insulin secretion by pancreatic islets [21, 22]. We speculated that Matrigel has a cytoprotective effect and confers particular benefits for transplanted islets, and that the islets could be protected against graft loss before the completion of neovascularization. The other reason for using it was that there were two types of Matrigel, containing growth factors or not (growth factor-reduced (GFR) Matrigel). Matrigel contains a number of angiogenic growth factors, and therefore represents a good model for evaluating the utility of growth factors in intramuscular transplantation compared with GFR Matrigel. We speculated that Matrigel (containing growth factors) could improve the engraftment of islets in muscle by overcoming the poor oxygen tension and neovascularization. The present study demonstrated the utility and mechanisms underlying the efficacy of the combination treatment of ECM and growth factors in intramuscular islet transplantation.

## Materials and Methods

### Ethical statement

All animal care and treatment procedures were performed in accordance with the institutional regulations of Tokohu University, Japan. The Institutional Animal Care Use Committee of the Tohoku University Graduate School of Medicine approved the experimental protocol used in the present study.

### Study design

We performed *in vitro* and *in vivo* experiments to evaluate the utility of ECM and growth factors in improving the efficacy of islet transplantation. In *in vitro* experiments, the residual rate, viability, and insulin releasing function in response to glucose stimulation of cultured islets were evaluated following culture in the presence of ECM and growth factors, or ECM only, or no treatment. Furthermore, islet protein expression levels, as a measure of signaling pathway activity, under the three culture conditions, were evaluated by Western blotting to elucidate the mechanisms underlying the effects of ECM and growth factors on cellular proliferation and cytoprotection.

In *in vivo* experiments, intramuscular islet transplantation was performed in diabetic mice. Islets were inserted into the muscular space of diabetic mice with ECM and growth factors, ECM only, or without any treatment. Transplantation outcomes were evaluated by BG levels, serum insulin levels, and glucose tolerance tests. Islet grafts were harvested from a proportion of transplanted mice for the histological assessment of islet engraftment; islet engraftment (insulin), apoptosis [TdT-mediated deoxyuridine triphosphate-biotin nick end labeling (TUNEL assay)], cellular growth (Ki67), and neovascularization (CD31) were evaluated.

### Animals

For all experiments, 9–12-week-old male BALB/C mice weighing 25–30 g (CLEA Japan, Tokyo) were used as donors and diabetic recipients. Animals were housed under pathogen-free conditions on a 12-h light cycle with free access to food and water.

### Islet isolation

Islet isolation was performed using a modified version of Gotoh’s method [23]. In brief, murine islets were isolated by collagenase digestion (collagenase V, Sigma-Aldrich, St. Louis, MO, USA), separated by Ficoll discontinuous gradients (Ficoll PM 400, Sigma-Aldrich), and then purified. Isolated islets were cultured at 37°C in 5% CO_2_/95% air overnight. Following culture, colonies between 100 and 200 μm in size [defined as islet equivalent (IEQ = 150 μm)] were isolated and used in all further experiments.

### Induction of DM in recipient mice

Streptozotocin (STZ, 150 mg/kg, Sigma-Aldrich) was administered via the tail vein of BALB/C mice. BG levels were measured using a BG meter (Ascensia Breeze, Bayer Health Care, Kita, Japan). Animals with BG levels greater than 350 mg/dl on two consecutive measurements were used for transplantation studies.

### Assessment of the efficacy of intramuscular and intraportal islet transplantation

Before assessing the utility of ECM and growth factors, we compared the efficacy of intramuscular transplantation and intraportal islet transplantation. Using the intramuscular transplant method, islets were infused into the left femoral muscle along the direction of the muscle fibers using 27G Sureshield™ Safety Winged Infusion Sets (TERUMO, Tokyo, Japan) with 300 μL RPMI1640 media. Using the intraportal transplant method, islets were infused into the liver via the portal vein (superior mesenteric vein at pancreas) using 27G Sureshield™ Safety Winged Infusion Sets with 300 μL RPMI1640 media. Syngeneic 100, 200, 300, and 500 IEQs were transplanted into the muscle or portal vein of transplant recipients (*n* = 20 per group). The transplant efficacy was evaluated according to the proportion of animals achieving normoglycemia at 28 days following transplantation. Normoglycemia was defined as BG levels <200 mg/dl at two consecutive measurements.

### Extracellular matrices

Two Matrigel preparations were used to compare the therapeutic effect of ECM with growth factors and ECM only. Matrigel™ (BD Matrigel Basement Membrane Matrix: BD Biosciences, San Diego, CA) was used as a model of ECM with growth factors. Matrigel™ is a solubilized basement membrane preparation extracted from Engelbreth–Holm–Swarm mouse sarcomas. The major component of Matrigel is laminin (approximately 60% of total ECM protein), collagen IV (approximately 30%), entactin/nidogen (approximately 7%), and heparan sulfate proteoglycan. Matrigel™ also contains a number of growth factors including epidermal growth factor (EGF), platelet-derived growth factor (PDGF), and insulin-like growth factor 1 (IGF-1) (Table 1).

**Table 1:** Growth factor concentrations in Matrigel and growth factor-reduced Matrigel. [24]

Growth factor	*Matrigel	GFR Matrigel
EGF (ng/ml)	**0.5–1.3**	<0.5
bFGF (pg/ml)	0–0.1	0–0.1
NGF (ng/ml)	<0.2	<0.2
PDGF (pg/ml)	**12**	<5
IGF-1 (ng/ml)	**15.6**	5
TGF-β (ng/ml)	2.3	1.7

*The average concentrations of growth factors in Matrigel are shown as multiple lots were used. GFR, growth factor reduced.

Matrigel contains physiological concentrations of growth factors. Growth factor-reduced (GFR) Matrigel has substantially reduced concentrations of growth factors and was used as a model of ECM without growth factors in the present study. The concentrations of growth factors contained in Matrigel and GFR Matrigel are shown in Table 1. All growth factors shown in Table 1 have been shown to have angiogenic potential [14, 25–30].

### *In vitro* assessment of residual rate, viability, and insulin-releasing function of cultured islets

The cytoprotective effect of ECM was evaluated by determining the residual rate, cell viability, and insulin-releasing function of cultured islets. As we described previously, isolated islets were divided into three groups: islets without any treatment as a negative control group (islets-only group), islets embedded in GFR Matrigel (GFR Matrigel group), and islets embedded in Matrigel (Matrigel group). Fifty islets from each group were cultured in RPMI1640 medium (Sigma-Aldrich) supplemented with 11 mM glucose, 10% fetal bovine serum, 1% penicillin-streptomycin solution (Pen-Strep, Invitrogen, Carlsbad, CA), and 10 mM nicotinamide (Sigma-Aldrich) for 96 h. Islets were cultured in Matrigel and GFR Matrigel according to the following methods: (1) 1 ml Matrigel or GFR Matrigel was liquefied in 60 mm Petri dish at 4°C, (2) 50 islets were poured into the liquid with a small amount of cold RPMI1640 media (within 30 μL), (3) and embedded into the Matrigel solutions and gelated by warming at 37°C for 2–3 minutes, and (4) 5 ml warm RPMI1640 media (37°C) were then poured into the Petri dish.

The residual rate at 96 h after plating, an indicator of the stability of islets in the culture conditions, was calculated as the number of islets observed at 96 h per number of islets observed at the start of culture.

Islet cell viability at 96 h was defined as the percentage of viable cells (stained by SYTO-Green 11; Life Technologies Japan, Tokyo, Japan) per total number of viable and dead cells (stained by ethidium bromide; Sigma-Aldrich). Three islets per isolation were used for viability calculations. The average viability was calculated at time point.

Insulin-releasing function in response to glucose stimulation was examined by static incubation of cultured islets in low- and high-concentration glucose solutions. In detail, islets embedded in either Matrigel or GFR Matrigel and cultured in RPMI1640 medium for 96 h were extracted by depolymerization with 5 ml cell recovery solution at 4°C for 2 hs (BD Cell Recovery Solution; BD Biosciences). Islets in the islets-only group were also incubated with cell recovery solution to ensure similar treatment between groups. Ten islets from each group were plated onto cell culture inserts (BD Falcon) in 24-well plates and incubated in 1 ml of RPMI1640 medium containing low glucose (3.3 mM) for 1 h as a preincubation step. Next, islets were incubated in media containing 3.3 mM glucose for 1 h (termed sample “L”) and then media containing 16.7 mM glucose for 1 h (termed sample “H”). Samples were collected to allow measurement of insulin secretion. Released insulin was measured using insulin enzyme-linked immunosorbent assay kits (Shibayagi Co., Shibukawa, Japan). The ratio of insulin released between culture in sample H and sample L was calculated as the stimulation index (SI).

### Western immunoblotting

Focal adhesion kinase (FAK) phosphorylation and signaling downstream of mitogen-activated protein kinase / extracellular signal-regulated kinase 1/2 (MAPK/Erk) and phosphoinositide 3-kinase / Akt (PI3K/Akt) pathways via integrins were examined by Western immunoblotting. A total of 200 islets per group were cultured in fetal bovine serum containing RPMI1640 medium for 7 days prior to assessment. Following culture in RPMI1640 medium, islets were extracted by depolymerization of Matrigel and GFR Matrigel using cell recovery solution. To ensure treatments were similar between groups, the islets-only group was also incubated with cell recovery solution.

Protein concentrations in islet cell lysates were determined using bicinchroninic acid (BCA) protein assay kits (Thermo Fisher Scientific, Yokohama, Japan) with bovine serum albumin used as a standard control. Protein was extracted by cell lysis buffer, which consisted of 20 mM Tris-hydrochloric acid (HCl) (pH 7.5), 150 mM sodium chloride (NaCl), 1 mM disodium ethylenediaminotetraacetate (Na_2_EDTA), 1 mM ethylene glycol tetraacetic acid (EGTA), 1% Triton, 2.5 mM sodium pyrophosphate, 1 mM beta-glycerophosphate, 1 mM sodium orthovanadate (Na_3_VO_4_), 1 μg/ml leupeptin, included in the kit. Cell lysate proteins were fractionated by 4%– 15% sodium dodecyl sulfate (SDS)-polyacrylamide gradient gels with Tris/glycine/SDS buffer (Bio-Rad, Hercules, CA) and transferred to polyvinylidene difluoride membranes (Bio-Rad) using the Trans-Blot Turbo Blotting System (Bio-Rad). Primary antibodies against Tyr397 phospho-FAK, total FAK, Ser473 phospho-Akt, total Akt, Thr202/Tyr204 Phospho-p44/42 MAPK, total Erk1/2, and glyceraldehyde phosphate dehydrogenase, and anti-rabbit IgG horseradish peroxidase-conjugated secondary antibody were purchased from Cell Signaling Technology (Beverly, MA). Blocking conditions and antibody concentrations were used according to manufacturers’ instructions. Immunoblot signals were enhanced by chemiluminescence using Clarity Western ECL Substrate (Bio-Rad). Protein bands were observed using an ImageQuant LAS 4000 mini biomolecular imager (GE Healthcare, Buckinghamshire, UK).

### Assessment of the utility of ECM in islet transplantation

Two hundred islets from the islets-only group, Matrigel group, or GFR Matrigel group were transplanted into the left femur muscle of diabetic recipient mice. Islets in the Matrigel or GFR Matrigel group were mixed with liquefied 300 μL Matrigel or GFR Matrigel, respectively, and infused into femur muscle along the direction of the muscle fibers using 27G Sureshield™ Safety Winged Infusion Sets (TERUMO, Tokyo, Japan). Islets in the islets-only group were also infused into muscle in the same manner as the other two groups with 300 μL RPMI1640 media. Transplantation outcomes were evaluated according to changes in BG levels, serum insulin levels, and glucose tolerance tests. BG levels were measured at postoperative days (PODs) 0, 1, 2, 3, 5, 7, 10, 14, 17, 21, 24, and 28. Serum samples for the measurement of insulin levels were taken on PODs 0, 3, 7, 14, and 28. Serum insulin levels were measured using mouse insulin enzyme-linked immunosorbent assay kits. BG measurements and the collection of serum samples were performed on nonfasting mice between 8 and 10 am. Intraperitoneal glucose tolerance tests (IPGTTs) were performed on POD 29 after the mice had been fasted for 8 h. BG levels were measured at 0, 30, 60, 90, and 120 min after intraperitoneal injection of a 2 g/kg glucose solution. IPGTTs were evaluated by calculating change in the area under the curve (AUC) of glucose concentrations.

BG levels, serum insulin levels, and IPGTT AUC values were further compared between the Matrigel group and mice undergoing intraportal islet transplantation to evaluate transplant efficacy in the Matrigel group and provide a comparison for the intramuscular islet transplant model. Equal numbers of islets (200 IEQs) were transplanted in both groups.

### Histological examinations

Graft tissues for histological assessment were harvested from a number of mice in the islets-only group (including intraportally transplanted mice), the GFR Matrigel group, and the Matrigel group at PODs 3 and 14.

All the tissues were fixed by 10% formalin, embedded by paraffin, and sectioned at 5 μm. Histological specimens were stained with hematoxylin and eosin to assess cellular changes. Insulin and Ki67 immunohistochemistry were performed to identify islets and proliferating cells, respectively, in islets at POD 3. CD31 immunohistochemistry was performed to assess revascularization. Primary antibodies used were mouse anti-insulin antibody (clone K36AC10, Sigma-Aldrich) diluted 1:1000, rabbit anti-Ki67 antibody (clone SP6, Nichirei Bioscience, Tokyo) diluted 1:200, and goat anti-CD31 antibody (Santa Cruz Biotechnology, Santa Cruz, CA) diluted 1:800. Following incubation with biotinylated secondary IgG antibodies, binding was visualized using a peroxidase substrate solution containing 3,3′-diaminobenzidine (Dako, Glostrup, Denmark).

Islet apoptosis was evaluated using a TUNEL assay using peroxidase in situ apoptosis detection kits (Millipore, Billerica, MA) based on a Discovery XT system. Following proteinase treatment and inactivation of endogenous peroxidase, islets were incubated with TdT enzyme for 32 min at 37°C, further incubated with peroxidase-conjugated anti-digoxigenin (DIG) antibody (Millipore), and visualized with diaminobenzidine.

All histological assessments, except insulin immunohistochemistry, were quantified. The proportion of islets cells undergoing apoptosis was defined as the ratio of TUNEL-positive cells to the total number of islet cells. Assessments were performed using at least 20 islets per group. The proportion of proliferating islet cells was defined as the ratio of Ki67-positive cells to the total number of islet cells. Over 32 islets per group were used for proliferation assessments. Revascularization of transplanted islets was evaluated by quantifying the number of CD31-positive microvessels directly contiguous with transplanted islets. Over 21 views at 400× magnification per group were used for the assessment of revascularization. Revascularization per unit area in transplanted islets was evaluated by quantifying the number of CD31-positive microvessels in individual islets. Over 17 islets per group were used for the assessment of islet revascularization.

### Statistical analyses

BG levels, the proportion of STZ-treated mice achieving normoglycemia, serum insulin levels, and AUC of change in glucose concentrations during IPGTT were compared among the islets-only, GFR Matrigel, and Matrigel groups. Differences in BG levels, serum insulin levels, and IPGTT AUC values were evaluated by two-way repeated measures analysis of variance. The proportion of STZ-treated mice achieving normoglycemia was compared between groups using the Kaplan–Meier method and the log-rank test. Islet residual rates, islet cell viability, stimulation indices, and quantitative histological assessments were compared among the islets-only, GFR Matrigel, and Matrigel groups using Dunnett’s test. All data are presented as means ± standard errors of the mean. *P*-value of <0.05 was considered statistically significant.

## Results

### Efficacy of intramuscular and intraportal islet transplantation

**Fig 1:**
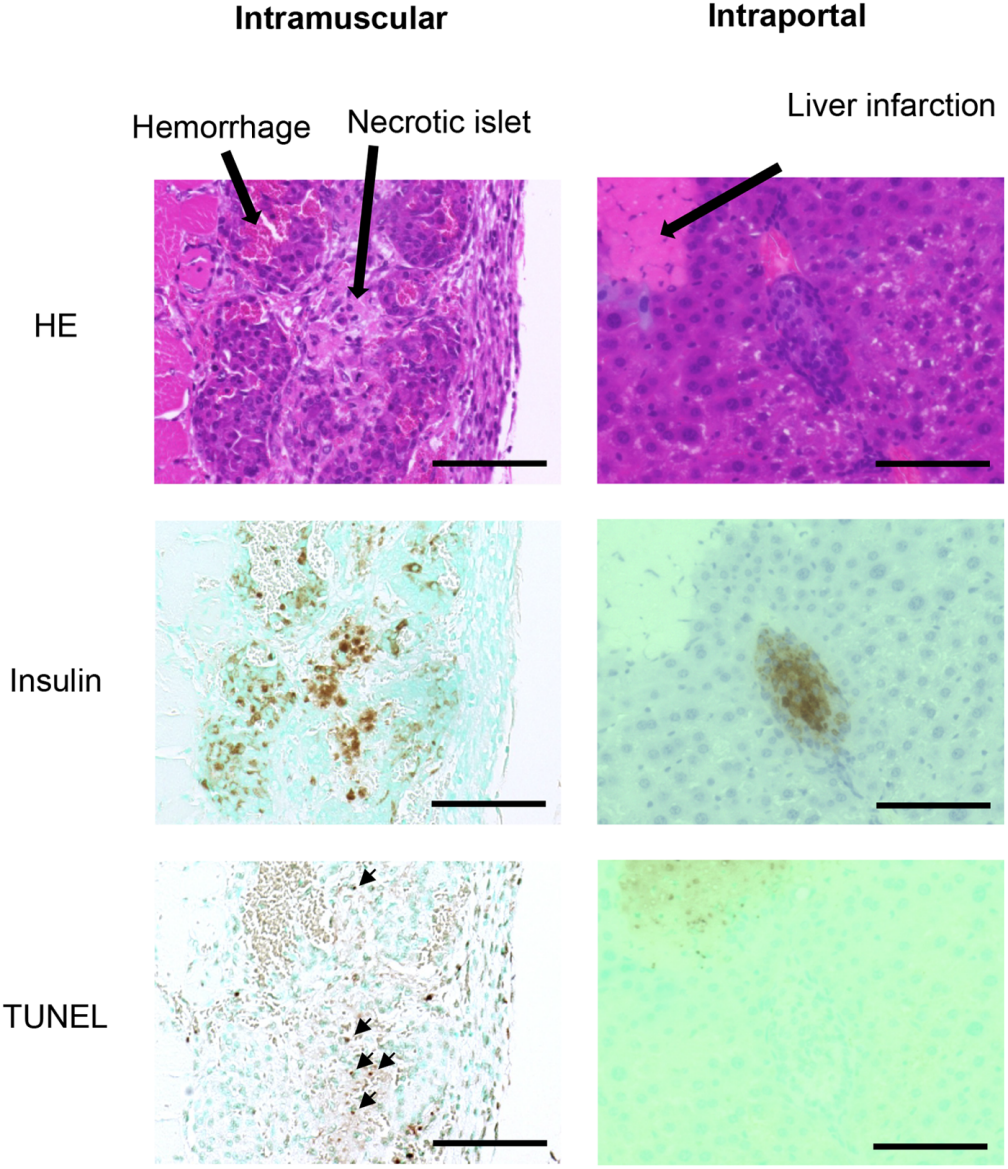
Condition of intraportally and intramuscularly transplanted islets at POD3. Substantial inflammatory infiltrates adjacent to intramuscularly transplanted islets with hemorrhage around islets were observed. Transplanted islets were observed as clustered aggregates with necrotic and apoptotic islet cells at the center of clusters. Necrotic and apoptotic cells were rarely observed following intrahepatic islet transplantation. Scale bar, 100 μm.

The proportion of mice achieving normoglycemia following intramuscular and intraportal transplantations is shown in Table 2. Normoglycemia was induced with the intraportal transplantation of 200 islets; however, a minimum of 300 intramuscularly transplanted islets was required to induce the same effect. Histological findings at POD 3 revealed substantial inflammatory infiltrates adjacent to intramuscularly transplanted islets. Transplanted islets were observed as clustered aggregates with necrotic cells, observed as cells with eosinophilic cytoplasm and nuclear shedding, and apoptotic cells observed at the center of clustered aggregates. Conversely, while a number of hepatic infarctions were observed following intraportal islet transplantation, necrotic or apoptotic islets were rarely observed following intraportal transplantation (Fig 1).

**Table 2:** Proportion of mice achieving normoglycemia following islet transplantation and MD50 values for each transplant site.

	Proportion of mice achieving normoglycemia (%)			
	100 IEQs	200 IEQs	300 IEQs	500 IEQs^a^
	(n = 5)	(n = 5)	(n = 5)	(n = 5)
**Intraportal**	0	**20**	**60**	**100**
**Intramuscular**	0	0	**40**	**60**

^a^IEQ, islet equivalent.

### Effect of ECM on islet function *in vitro*

**Fig 2:**
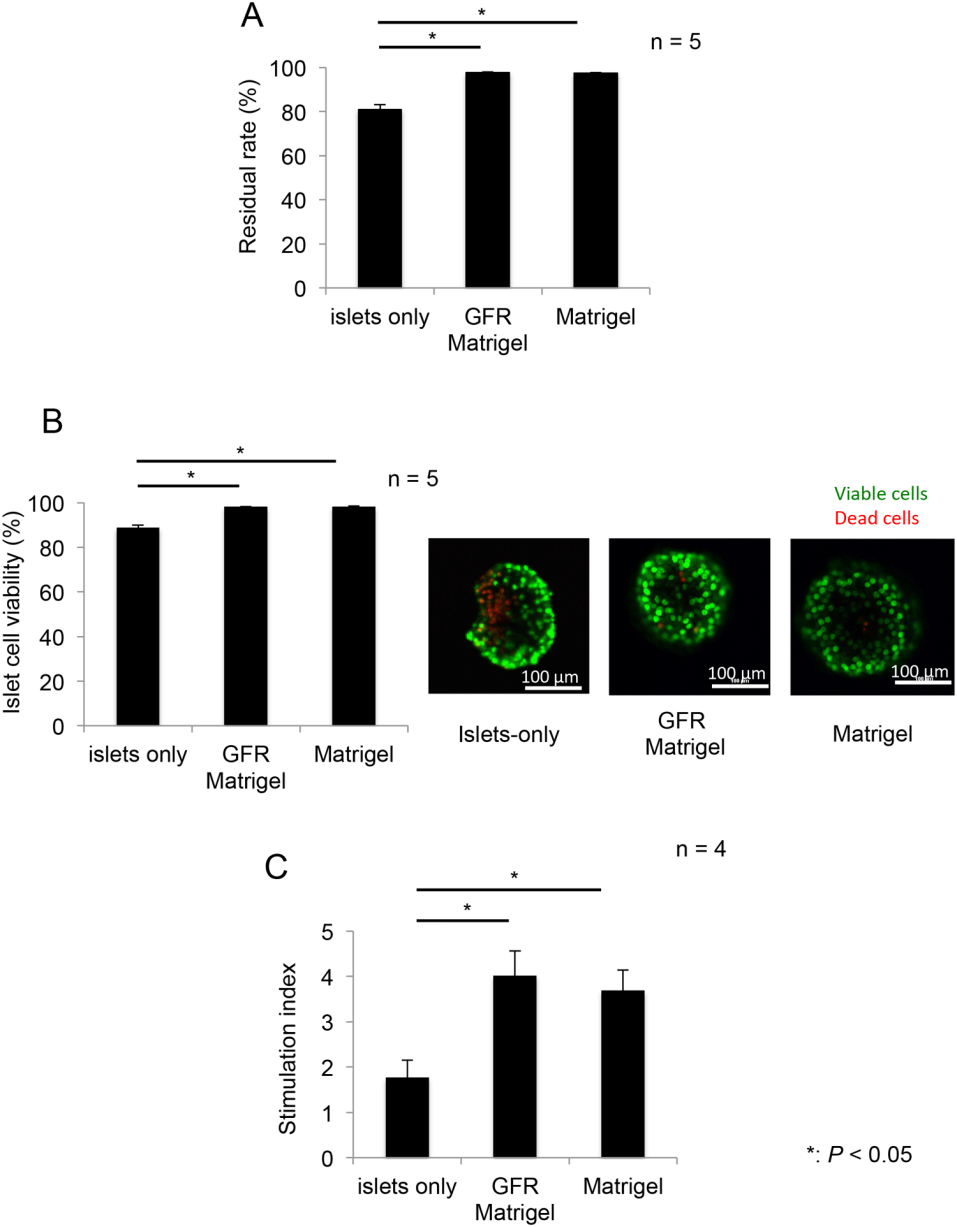
Islet residual rate, viability, and stimulation index following culture with or without Matrigel. Islets were cultured for 96 h with or without Matrigel before the assessment of residual rate, viability, and the ratio of insulin released in response to stimulation by high and low concentration glucose solutions (SI). (**A**) The residual rate of islets in the GFR Matrigel or Matrigel groups was significantly higher than that in the islets-only group. (**B**) Islet cell viability in GFR Matrigel or Matrigel groups was significantly higher than that in the islets-only group. Right figures; green: viable cells, red: dead cells. Scale bar, 100 μm. (**C**) The SI was significantly higher in the GFR Matrigel and Matrigel groups compared with the islets-only group. *P < 0.05. GFR, growth factor reduced; SI, stimulation index.

Islet residual rates (*n* = 5 per group) in the GFR Matrigel and Matrigel groups were significantly higher than those in the islets-only group (islets-only, 80.9% ± 2.19%; GFR Matrigel, 97.6% ± 90.36%; Matrigel, 97.5% ± 0.35%; *P* = 0.0001 for islets-only vs. GFR Matrigel; *P* = 0.0002 for islets-only vs. Matrigel; Fig 2A). Islet cell viability (*n* = 5 per group) in the GFR Matrigel and Matrigel groups was significantly higher than that in the islets-only group (islets-only, 88.7% ± 1.16%; GFR Matrigel, 98.1% ± 0.22%; Matrigel, 98.2% ± 0.28%; *P* = 0.0001 for islets-only vs. GFR Matrigel; *P* = 0.0001 for islets-only vs. Matrigel; Fig 2B). The SI (*n* = 4) was significantly higher in the GFR Matrigel and Matrigel groups compared with the islets-only group (islets-only, 1.77 ± 0.39; GFR Matrigel, 4.02 ± 0.54; Matrigel, 3.69 ± 0.45; *P* = 0.026 for islets-only vs. GFR Matrigel; *P* = 0.031 for islets-only vs. Matrigel; Fig 2C). These data demonstrate the utility of ECM (regardless of growth factors) in reducing islet injury and preserving islet endocrine function during *in vitro* culture conditions.

**Fig 3:**
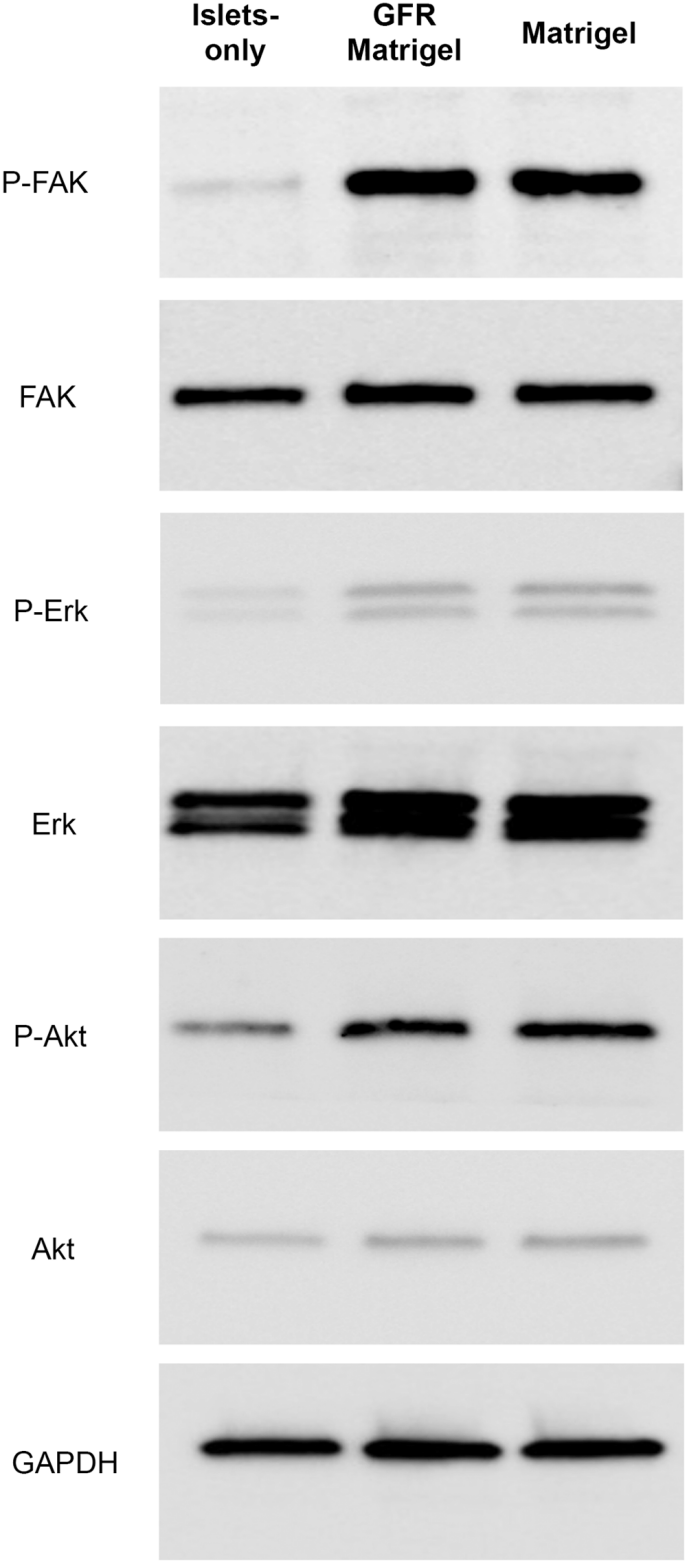
Intracellular signaling activity in islets cultured in Matrigel. FAK, Erk, and Akt protein levels in islets were evaluated by Western immunoblotting to assess the effect of Matrigel on cellular growth and cytoprotection. Cell lysates obtained from the islets-only, Matrigel, and GFR Matrigel groups were subjected to SDS-PAGE and Western immunoblotting using the indicated antibodies. Glyceraldehyde, phosphate dehydrogenase was used as the loading control. Phosphorylated FAK, Erk, and Akt were strongly expressed in the Matrigel and GFR Matrigel groups compared with the islets-only group. Erk, extracellular signal-regulated kinase; FAK, focal adhesion kinase; GFR, growth factor-reduced; SDS, sodium dodecyl sulfate.

Regarding intracellular signaling activity in islets cultured in ECM, increased expression of phosphorylated FAK, Erk, and Akt was clearly demonstrated in the Matrigel and GFR Matrigel groups compared with the islets-only group (Fig 3). These data indicate culture in ECM stimulates MAPK/Erk and PI3K/Akt pathways, contributing to the promotion of islet cellular proliferation and survival.

### ECM containing growth factors contributes to improved endocrine function of intramuscularly transplanted islets

**Fig 4:**
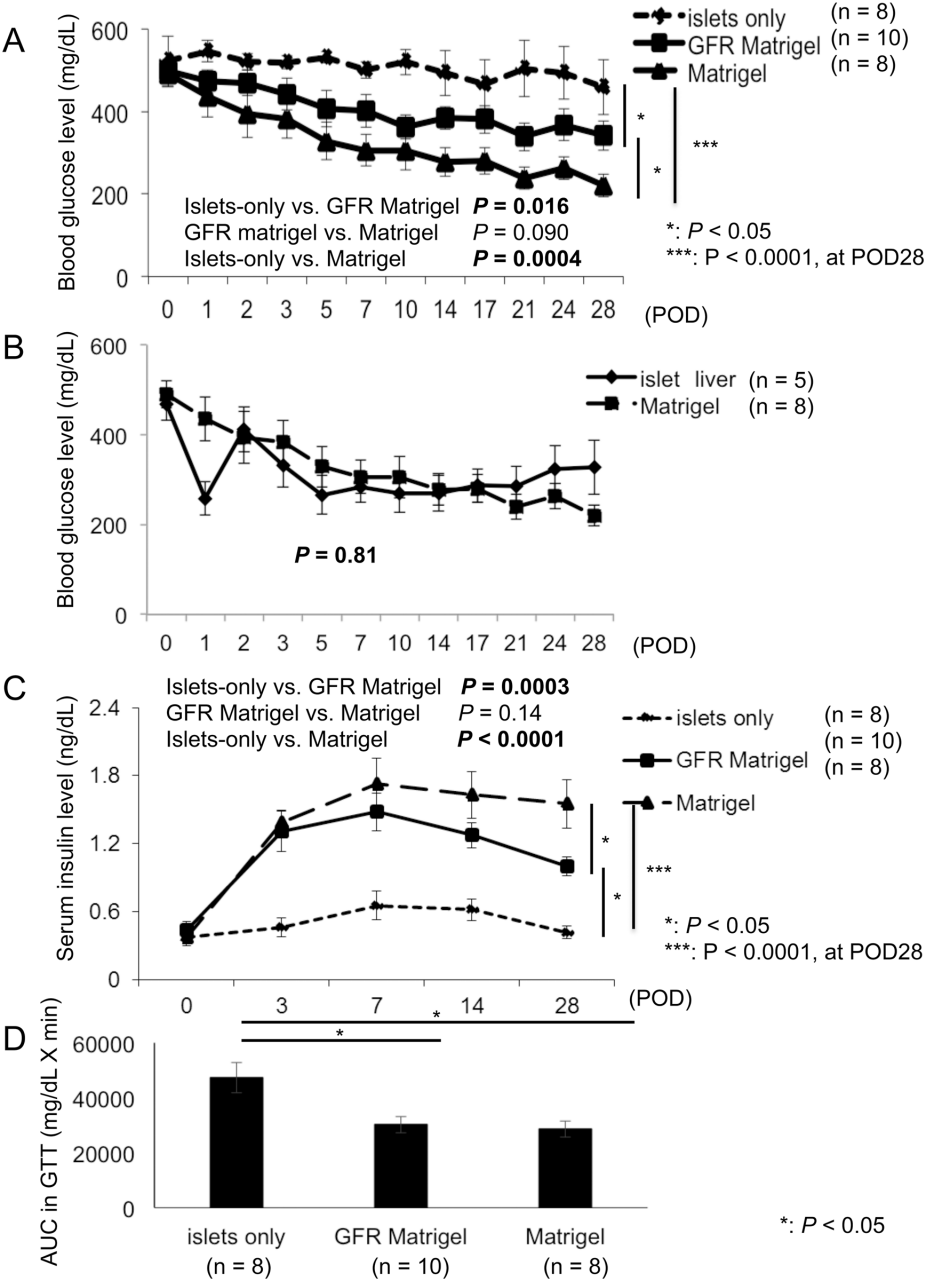
Changes in the blood glucose concentrations, serum insulin level, and IPGTT following transplantation. Transplantation outcomes were evaluated by the measurement of BG and serum insulin levels and GTTs. (**A**) BG levels were significantly improved in islets embedded in ECM (both Matrigel and GFR Matrigel groups) compared with the islets-only group. BG levels were significantly lower in the Matrigel group compared with the GFR Matrigel group at POD 28. (**B**) Comparison of changes in blood glucose concentrations between intramuscular transplantation in the Matrigel group and the intraportal transplantation group. No significant differences were observed in changes in blood glucose concentration between intramuscular transplantation in the Matrigel group and the intrahepatic transplantation group. (**C**) Serum insulin levels were significantly increased in the GFR Matrigel and Matrigel groups compared with the islets-only group, and levels in the Matrigel were significantly higher than those in the GFR Matrigel group at POD 28. (**D**) IPGTT AUC values in both the GFR Matrigel and Matrigel groups were significantly lower than those in the islets-only group. AUC, area under the curve; BG, blood glucose; ECM, extracellular matrix; GFR, growth factor reduced; GTT, glucose tolerance test; IPGTT, intraperitoneal glucose tolerance test; POD, postoperative day.

Following syngeneic intramuscular transplantation, BG levels were significantly improved following transplantation of islets embedded in ECM (both Matrigel and GFR Matrigel) compared with the islets-only group (*P* = 0.0004 and 0.016; Fig 4A). The difference between Matrigel and GFR Matrigel groups gradually increased after transplantation and became prominent from POD 7. At POD 28, BG levels were significantly lower in the Matrigel group compared with the GFR Matrigel group (islets-only, 459.3 ± 66.4 mg/dl; GFR Matrigel, 342.2 ± 35.9 mg/dl; Matrigel, 220.3 ± 27.0 mg/dl; *P* = 0.043 for islets-only vs. GFR Matrigel; *P* < 0.0001 for islets-only vs. Matrigel; *P* = 0.022 for GFR Matrigel vs. Matrigel; Fig 4A). The proportion of mice achieving normoglycemia following transplantation was 0% in the islets-only group, 20% in the GFR Matrigel group, and 50% in the Matrigel group (*P* = 0.19 for islets-only vs. GFR Matrigel; *P* = 0.025 for islets-only vs. Matrigel; *P* = 0.16 for GFR Matrigel vs. Matrigel). The reason why all the groups did not reach under 200 mg/dL in BG level was because some mice that failed to reach normoglycemia had high glucose levels. The proportion of mice in the Matrigel group achieving normoglycemia following intramuscular transplantation was equal to or greater than in mice receiving intraportal transplantation of islets (Fig 4A; Table 2), with no difference observed in the change in BG concentrations between intramuscular transplantation in the Matrigel group and the intrahepatic transplantation group (*P* = 0.81; Fig 4B).

Serum insulin levels were significantly increased in the Matrigel and GFR Matrigel groups compared with the islets-only group (*P* < 0.0001 and = 0.0003; Fig 4C). There was no difference between the Matrigel and GFR Matrigel groups until POD 3, but a difference could be seen at POD 7 and became prominent after that. At POD 28, serum insulin levels in the Matrigel group were significantly higher than those in the GFR Matrigel group (islets-only, 0.42 ± 0.06 ng/dl; GFR Matrigel, 1.00 ± 0.08 ng/dl; Matrigel, 1.55 ± 0.21 ng/dl; *P* < 0.0001 for islets-only vs. GFR Matrigel; *P* = 0.0003 for islets-only vs. Matrigel; *P* = 0.026 for GFR Matrigel vs. Matrigel; Fig 4C). IPGTT AUC values in both the GFR Matrigel and Matrigel groups were significantly lower than those in the islets-only group (islets-only, 47,288 ± 5,481 mg/dl × min; GFR Matrigel, 30,201 ± 3,063 mg/dl × min; Matrigel, 28,648 ± 2,957 mg/dl × min; *P* = 0.016 for islets-only vs. GFR Matrigel; *P* = 0.014 for islets-only vs. Matrigel; Fig 4D).

### ECM containing growth factors contributes to improved engraftment of intramuscularly transplanted islets by reducing apoptosis and promoting proliferation and angiogenesis

**Fig 5:**
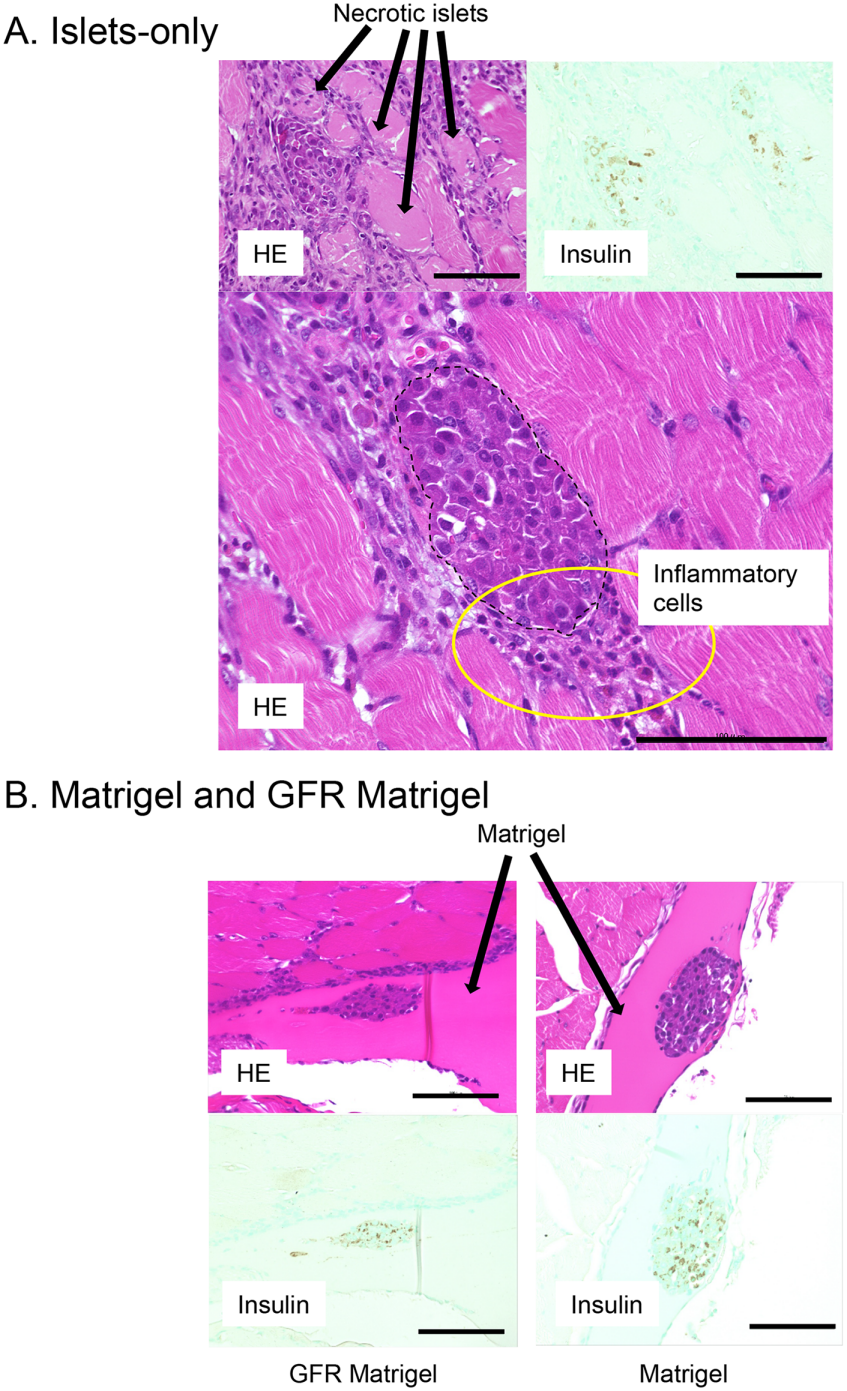
Intramuscular transplanted islets at POD 3. A. A large number of inflammatory infiltrates (yellow circle) were observed surrounding islets with a number of islets found to have underwent necrosis in the islets-only group. B. Islets in the GFR Matrigel and Matrigel groups were separated from surrounding inflammatory cells by extracellular matrix. Few necrotic islets were observed in the Matrigel groups. Upper panel, hematoxylin and eosin. Lower panel, insulin immunohistochemistry. Scale bar, 100 μm. POD, postoperative day.

Fig 5 shows HE and insulin immunohistochemistry in intramuscularly transplanted islets at POD 3. Substantial inflammatory infiltrates were observed adjacent to islets with a number of necrotic islets in the islets-only group (Fig 5A), whereas islets in the GFR Matrigel and Matrigel groups were separated from inflammatory cells by ECM. Few necrotic islets were seen in the GFR Matrigel and Matrigel groups (Fig 5B).

**Fig 6:**
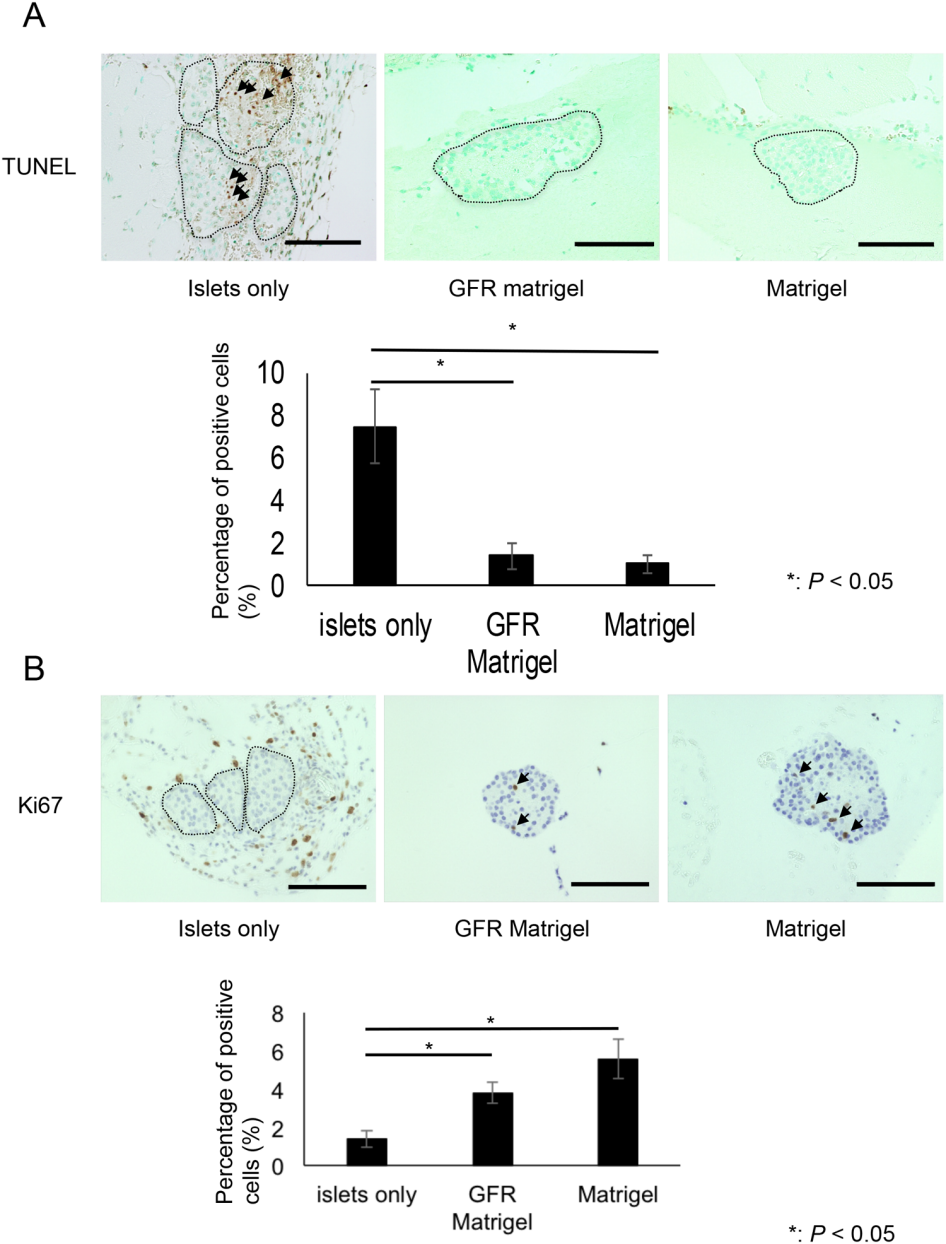
TUNEL and Ki67 immunohistochemistry of intramuscularly transplanted islets at POD 3. (**A**) The proportion of TUNEL-positive cells (arrows) per islet was significantly lower in the GFR Matrigel and Matrigel groups compared with the islets-only group. The proportion of TUNEL-positive apoptotic cells was 7.50 ± 1.79 in the islets-only group, 1.37 ± 0.65 in the GFR Matrigel group, and 1.01 ± 0.41 in the Matrigel group (*P* = 0.0014 for islets-only vs. GFR Matrigel; *P* = 0.0024 for islets-only vs. Matrigel). (**B**) The proportion of Ki67-positive cells (arrows) per islet was significantly higher in the GFR Matrigel and Matrigel groups than that in the islets-only group. The proportion of Ki67-positive cells per islet was 1.38 ± 0.43 in the islets-only group, 3.80 ± 0.55 in the GFR Matrigel group, and 5.58 ± 1.02 in the Matrigel group (*P* = 0.001 for islets-only vs. GFR Matrigel; *P* = 0.00025 for islets-only vs. Matrigel). Scale bar, 100 μm GFR, growth factor reduced; TUNEL, TdT-mediated deoxyuridine triphosphate-biotin nick end labeling.

The proportion of TUNEL-positive cells per islet was significantly higher in the islets-only group compared with the Matrigel and GFR Matrigel groups. The proportion of apoptotic cells was 7.50% ± 1.79% in the islets-only group, 1.37% ± 0.65% in the GFR Matrigel group, and 1.01% ± 0.41% in the Matrigel group (*P* = 0.0014 for islets-only vs. GFR Matrigel; *P* = 0.0024 for islets-only vs. Matrigel; Fig 6A). In addition, the proportion of Ki67-positive cells per islet was significantly higher in the GFR Matrigel and Matrigel groups compared with the islets-only group (islets-only group, 1.38% ± 0.43%; GFR Matrigel group, 3.80% ± 0.55%; Matrigel group, 5.58% ± 1.02%; *P* = 0.001 for islets-only vs. GFR Matrigel; *P* = 0.00025 for islets-only vs. Matrigel; Fig 6B). These data indicate ECM inhibits islet cell apoptosis and promotes proliferation of islet cells.

**Fig 7:**
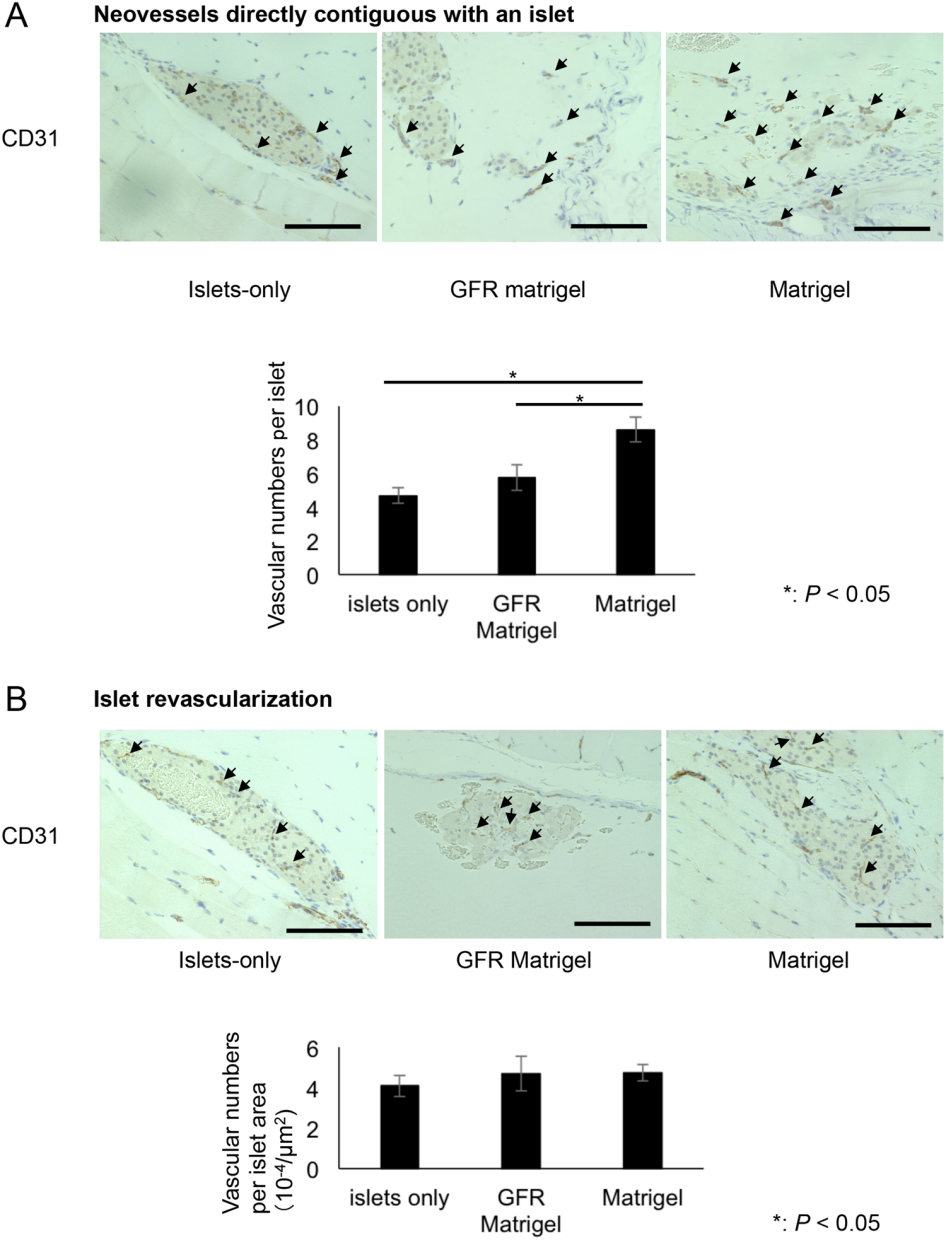
CD31 staining of intramuscularly transplanted islets at POD 14. (**A**) Assessment of neovessels directly contiguous with islets. The number of CD31-positive microvessels (arrows) adjacent to islets was significantly higher in the Matrigel group than in the islets-only and GFR Matrigel groups. The number of microvessels adjacent to islets was 4.68 ± 0.47 in the islets-only group, 5.75 ± 0.75 in the Matrigel group, and 8.61 ± 0.75 in the GFR Matrigel group (*P* = 0.0001 for islets-only vs. Matrigel; *P* = 0.012 for GFR Matrigel vs. Matrigel). (**B**) Assessment of islet revascularization. No significant difference in the number of microvessels was observed among the three groups. Scale bar, 100 μm GFR, growth factor reduced; POD, postoperative day.

Regarding revascularization, the number of CD31-positive microvessels directly contiguous with islets was significantly higher in the Matrigel group compared with the islets-only and GFR Matrigel groups (islets-only, 4.68 ± 0.47 per islet; GFR Matrigel, 5.75 ± 0.75 per islet; Matrigel, 8.61 ± 0.75 per islet; *P* = 0.0001 for islets-only vs. Matrigel; *P* = 0.012 for GFR Matrigel vs. Matrigel; Fig 7A). No significant difference in the number of vessels per islet was observed between the three groups (islets-only, 4.08 ± 0.53 × 10^−4^/μm^2^; GFR Matrigel, 4.71 ± 0.86 × 10^−4^/μm^2^; Matrigel, 4.74 ± 0.40 × 10^−4^/μm^2^; Fig 7B). These data indicate the growth factors contained in Matrigel specifically induce revascularization at the site of islet transplantation.

## Discussion

The muscle is an ideal candidate transplant site for islets as it has been shown to be associated with decreased technical difficulty in surgical procedure, greater ease of monitoring, and reduced rates of instant blood-mediated inflammatory reaction in several animal studies and human clinical trials [31–33]. Subsequently, Lund *et al*. examined the transplant efficacy of intramuscular transplantation compared with intraportal transplantation in a rat model and unexpectedly found decreased efficacy compared with intraportal transplantation [34]. In contrast, Christoffersson *et al*. demonstrated the efficacy of intramuscular islet transplantation was superior to that of intraportal transplantation, and the infiltration of neutrophils during the early stages of transplantation was important for revascularization [35]. Our data demonstrated increased numbers of islet are required to achieve normoglycemia with the use of intramuscular transplantation compared with intraportal transplantation and observed greater islet injury following intramuscular transplantation compared with intraportal transplantation. Svensson *et al*. demonstrated increased revascularization of islets transplanted into muscle tissue compared with intrahepatic transplantation. Further, oxygen levels in intramuscularly transplanted islets were only slightly lower than those in native pancreatic islets; however, the authors also observed greater frequencies of extensive fibrosis and central graft necrosis in intramuscular transplanted islets, particularly when islets were transplanted as clusters [36]. Another possible reason for the poor transplant efficacy in intramuscular transplantation is the adverse events induced by some cytokines, which are derived from each organ, like hepatokines (liver) and myokines (muscle). For example, selenoprotein P and leukocyte cell-derived chemotaxin 2 (LECT2), which are known as hepatokines, decrease insulin sensitivity in skeletal muscle [37, 38]. Interleukin (IL-) 6, which is known as a myokine, is a representative inflammatory cytokine [39]. Our previous study showed that the serum IL-6 level increased after intramuscular islet transplantation and it decreased after mesenchymal stem cell co-transplantation [40]. Our data in this study showed that many intramuscular transplanted islets were damaged by an inflammatory reaction. These changes might be affected by IL-6 derived from muscle at the transplant site. Thus, improvements in the efficacy of intramuscular islet transplantation are essential to overcome this limitation and allow the application of this approach to clinical settings.

One of the strategies that have demonstrated utility in improving intramuscular islet transplantation is the prevention of early loss of transplanted islets through improved engraftment efficiency. In accordance with this strategy, many groups have evaluated the utility of ECM in improving engraftment efficiency. A number of groups have demonstrated the utility of ECM in improving insulin-releasing function of transplanted islets [41, 42], improving revascularization, and preserving islet morphology [16]. In a transplant model, Bharat *et al*. demonstrated successful subcutaneous islet transplantation using ECM [43]. As with ECM, many growth factors, including VEGF [44], fibroblast growth factor (FGF) [45], or islet-1 (Isl1) [46], have also been studied for neovascularization and rich engraftment in islet transplantation. Thus, we attempted to demonstrate the utility and mechanisms underlying the efficacy of combination treatment of ECM and growth factors in intramuscular islet transplantation in this study. These findings may inform the development of future clinical islet transplantation techniques incorporating combination treatment of cultured islets with ECM and growth factors.

First, we observed the viability and insulin-releasing function of cultured islets were preserved in both Matrigel and GFR Matrigel groups. These findings indicate ECM prevents cultured islet injury and contributes to the maintenance of islet function. Second, the endocrine function in mice transplanted with islets cultured with ECM (both Matrigel and GFR Matrigel) was significantly improved compared with mice transplanted with islets-only. We propose the improvements in islet engraftment efficiency and endocrine function underlie the observed improvement in transplant efficacy.

ECM improves transplant efficacy through local immunosuppressive effects. As Lund *et al*. demonstrated, increased local inflammation in response to transplantation is a significant limitation of muscle as a transplant site. A previous study demonstrated intramuscular islet transplantation led to upregulation of pro-inflammatory cytokines including IL-6 and IL-8 [34]. Our data also indicated intramuscularly transplanted islets were injured by local inflammation in the islets-only group at POD 3. Local inflammation was shown to be reduced by the use of ECM (Matrigel and GFR Matrigel groups) by preventing physical contact between islets and inflammatory cells. Furthermore, both Matrigel and GFR Matrigel contain TGF-β, a potent anti-inflammatory cytokine [47–49]. The anti-inflammatory effects of ECM are thought to be mediated by the prevention of physical contact between islets and inflammatory cells and secreted inflammatory cytokines. Further, ECM is thought to prevent necrosis of transplanted islets. Our data demonstrate intramuscularly transplanted islets in the islets-only group tended to aggregate as clusters leading to central necrosis. Central cluster necrosis is likely due to increased hypoxia and ischemia compared with peripheral regions. Transplanted islets in the Matrigel and GFR Matrigel groups were prevented from forming clusters and were able to engraft as individual islets, thus preventing central necrosis. This effect of ECM in preventing cluster formation also led to inhibition of apoptosis. In addition, proliferation of β cells was significantly increased in both Matrigel and GFR Matrigel groups. Early islet loss may have been prevented by these beneficial effects of ECM.

The BG levels were lower, and serum insulin levels were higher, in the GFR Matrigel and Matrigel groups compared with the islets-only group during the study period. The difference in the BG level between the Matrigel and GFR Matrigel groups gradually increased after transplantation and became prominent from POD 7. On the other hand, no difference in serum insulin was seen until POD 3, but it gradually increased from POD 7. Finally, both were significantly prominent in the Matrigel group compared with the GFR Matrigel group at POD 28. The degree of early apoptosis and proliferation was similar between the Matrigel and GFR Matrigel groups, and many islets in the two groups survived by the useful effect of ECM at the early transplant stage (till POD 3), and thus the difference in the BG and serum insulin levels was not prominent at POD 3. After that, the islets in the Matrigel group were protected from graft loss by the angiogenesis induced by growth factors, while some of the islets in the GFR Matrigel group failed to engraft due to poorer angiogenesis at POD 7. As a result, a difference in the serum insulin level became apparent, and the difference in the BG level became prominent at POD 7. Neovascularization was completed by POD 14, and more islets succeeded to engraft in the Matrigel group than the GFR Matrigel group. Finally, the differences in both the BG and serum insulin levels were more prominent at POD 14 and 28 than before. In summary, transplanted islets, with potentially lower levels of early graft loss due to the use of ECM, were maintained for at least 3 days, with revascularization of the transplant site induced by growth factors contained in Matrigel (i.e., EGF, PDGF, IGF-1) likely having a significant contribution. These results indicate combination treatment with ECM and growth factors has efficacy in intramuscular islet transplantation.

The MAPK/Erk and PI3K/Akt intracellular signaling pathways play an important role in cellular growth and differentiation [50, 51]. It is known that ECM activates the MAPK/Erk and PI3K/Akt pathways through phosphorylation of FAK via integrins to promote cell survival and proliferation [52]. Recently, Hou and colleagues revealed that the apoptosis induced by hypoxia was inhibited through these pathways [53]. Western blotting in the present study demonstrated increased phosphorylation of FAK, Erk, and Akt in islets cultured with ECM (both Matrigel and GFR Matrigel). We suspect the observed inhibition of apoptosis induced by hypoxia and promotion of proliferation in islet cells treated with ECM was promoted by activation of these signaling pathways leading to increased islet cell viability. In addition, many of the growth factors contained in Matrigel may also have cytoprotective and cellular proliferative effects on β cells through activation of the MAPK/Erk or PI3/Akt pathways via tyrosine kinase receptors. Previous studies have demonstrated that EGF is involved in β-cell growth and prevention of β-cell death by stimulating the PI3-kinase/AKT signaling pathway [54, 55]. PDGF has also been shown to promote β-cell proliferation [56]. IGF-1 has been shown to prevent β-cell death resulting from oxidative stress [57, 58].

HIF-1α is an important marker for cellular proliferation survival in a hypoxic condition. Maillard and colleagues showed that HIF-1α was strongly expressed in islets cultured with ECM in a hypoxic condition, and activated caspase-3, an apoptotic marker, decreased in the islets [59]. Thus, we speculated that HIF-1α was strongly expressed in the islets in Matrigel and GFR Matrigel groups, and tried to prevent apoptosis and promote cellular proliferation.

We suspect that the combination of ECM and growth factors is more effective in activating these signaling pathways that contribute to the improved viability, function, and engraftment of islets. Especially, in the transplant study, many transplanted islets could successfully engraft because early graft loss was prevented and neovascularization occurred due to ECM and growth factors. It might be possible to achieve longer survival of engrafted islets by protecting them from adverse events, such as chronic inflammation, after transplantation [60]. Thus, it will be necessary to develop a novel ECM that can function for a longer time for the clinical setting.

## Conclusions

The results of the present study support the feasibility of intramuscular islet transplantation with the use of ECM and growth factors in clinical settings, even in situations where prevascularization is not feasible. Further studies are required to determine the most efficacious combinations of ECM and growth factors for islet transplantation in clinical settings.
